# The children’s nursing workforce in Kenya, Malawi, Uganda, South Africa and Zambia: generating an initial indication of the extent of the workforce and training activity

**DOI:** 10.1186/s12960-019-0366-4

**Published:** 2019-05-07

**Authors:** Natasha North, Maylene Shung-King, Minette Coetzee

**Affiliations:** 1 Child Nurse Practice Development Initiative, Department of Paediatrics and Child Health, Faculty of Health Sciences, University of Cape Town, Cape Town, South Africa; 2 Health Policy and Systems Division, Faculty of Health Sciences, School of Public Health and Family Medicine, University of Cape Town, Cape Town, South Africa; 3 Red Cross War Memorial Children’s Hospital, Klipfontein Road, Rondebosch, Cape Town, South Africa

**Keywords:** Nursing, Children, Africa, Workforce, Education, Training, Regulation, Health workforce, Paediatrics, Child

## Abstract

**Background:**

This study sought to identify, as far as possible, the extent of the specialist children’s nursing workforce in five selected African countries. Strengthening children’s nursing training has been recommended as a primary strategy to reduce the under-five mortality rate in African nations. However, information about the extent of the specialist children’s nursing workforce in this region is not routinely available. Developing an accurate depiction of the specialist children’s nursing workforce is a necessary step towards optimising children’s health service delivery.

**Methods:**

This study used a convergent parallel mixed methods design, incorporating quantitative (surveys) and qualitative (questionnaire and interview) components, to generate data addressing three related questions: how many children’s nurses are believed to be in practice nationally, how many such nurses are recorded on the national nursing register and how many children’s nurses are being produced through training annually.

**Results:**

Data provide insights into reported children’s nursing workforce capacity, training activity and national training output in the five countries. Findings suggest there are approximately 3728 children’s nurses across the five countries in this study, with the majority in South Africa. A total of 16 educational programmes leading to a qualification in paediatric nursing or child health nursing are offered by 10 institutions across the countries in this study, with Kenya, Malawi and Zambia having one institution each and South Africa hosting seven. Data suggest that existing human resources for health information systems do not currently produce adequate information regarding the children’s nursing workforce. Analysis of qualitative data elicited two themes: the role of children’s nurses and their position within health systems, and the capacity of HRH information systems to accurately reflect the specialist children’s nursing workforce.

**Conclusion:**

The data generated provide an initial indication of the size of the children’s nursing workforce in these five countries, as well as an overview of associated training activity. We hope that they can start to inform discussion about what would represent a viable and sustainable regional children’s nursing workforce for the future.

## Background

Nurses are the backbone of healthcare delivery in Africa, forming the largest part of the professional health workforce [1, 2]. Children aged 0–15 make up between 29 and 48% of the total population in the five countries studied (see Table 2) [3]. Children represent a high proportion of hospital admissions, with neonates in one South African study accounting for 40% of critical care bed occupation [4]. Outcomes for hospitalised children remain poor compared to higher resourced healthcare settings [5].

Strengthening children’s nursing training is recommended as a primary strategy to reduce the under-five mortality rate in African nations [6, 7]. Progress in postgraduate education and training in Africa has resulted in the presence within the workforce of a small cadre of nurses with specialised knowledge and skills relating to the care of sick babies and children, gained through additional qualifications [8]. These specialist children’s nurses are a scarce key resource in a region where more than 100 children per 1000 may die before the age of five [9] and where as many as 80% of nursing posts are vacant in some remote areas [7, 10].

Despite the potential importance of this cadre of nurses, there is a lack of accurate high-quality information about this emerging sector of the nursing workforce. Ideally, health management information systems would already be capable of monitoring the children’s nursing workforce in these countries. The reality is that children’s nurses in Africa are largely invisible to health information systems at the moment. Current data capabilities mean that most systems in lower income countries, including National Health Workforce Accounts, treat the nursing workforce as a single category. The absence of this information currently constrains the planning and creating of a viable and sustainable children’s nursing workforce. Developing an accurate depiction of the specialist registered children’s nursing workforce is a necessary step towards optimising children’s health service delivery and ultimately improving child health. This study sought to provide an initial estimation of the extent of the children’s nursing workforce in five selected African countries, by investigating the numbers of such nurses in practice and the scope of training activity, as a first step towards identifying what would represent a viable and sustainable children’s nursing workforce for the future.

### Terminology

The terms used to refer to the category of nurses considered in this study vary throughout the region. The widely understandable plain language description of ‘children’s nurse’ is used throughout this paper to refer to nurses who have undertaken post-basic training leading to a qualification as a specialist paediatric or child health nurse which is registerable with the relevant national nursing council.

## Methods

### Aim and design

This study aimed to quantify the extent of the children’s nursing workforce in five selected African countries. A convergent parallel mixed methods design was used, incorporating quantitative (surveys) and qualitative (questionnaire and interview) components. The methodology was selected to access both subjective and objective knowledge relating to the children’s nursing workforce, with the intention of maximising understanding and enabling corroboration between sources where existing factual information was known to be scarce [11, 12].

There were two key components of the study. Questionnaire surveys were administered to three groups of participants in order to elicit information about the number of children’s nurses respectively: in practice nationally, recorded on the professional register and being produced through training. Secondly, telephone interviews were scheduled with key informants in the final phase of data collection to supplement the information gathered. Interviewees had prior sight of the draft findings for their country and were asked to engage with the information to highlight inaccuracies or discrepancies and assist with corroboration.

Our study design included asking respondents and interviewees about the existence of recommendations for staffing norms specific to paediatrics. No specific recommendations were identified, and we are not aware of any made by the WHO or major global health organisations in relation to paediatric nurse ratios in LMICs.

### Setting and participants

This study extended to five African countries: Kenya, Malawi, Uganda, South Africa and Zambia. These countries were selected because they were believed to be actively training children’s nurses on the basis of knowledge obtained through sectoral engagement by the authors over the preceding 10 years. Documentary review revealed that very little information about the extent of the children’s nursing workforce in these countries was in the public domain. South Africa was the only country for which information relating to the numbers of children’s nurses on the national nursing register was available through published information. Current data capabilities mean that most health information systems in the countries studied, including National Health Workforce Accounts, treat the nursing workforce as a single category. This prior knowledge informed the approach to data collection. Our study design included asking respondents and interviewees about the existence of recommendations for staffing norms specific to paediatrics.

Expert elicitation sampling [13] was used, focusing on respondent characteristics that conferred a high level of familiarity with the children’s nursing workforce and/or access to records and relevant information. Criteria for inclusion were that the individual was a registrar of a national nursing council, the individual was a nursing educator at a training institution in the country or the individual held another leadership position within the national children’s nursing, paediatrics or child health communities. Expert sampling is recommended as an approach in situations where there is a lack of empirical knowledge and where uncertainty may be high [13]. The use of expert sampling was justified in the case of this investigation because of the objectively small size of the children’s nursing workforce in these countries and the lack of existing empirical data.

Two existing sources of information relevant to the question were identified, both of which were specific to South Africa. The record of the Child Nursing Educator Forum Nov. 2016 reported the number of children trained at 7/7 institutions in 2015. A separate study provided the number of children’s nurses trained at 5/7 institutions between 2012 and 2016, as well as information about the nature of this training activity (Chukwu, Shung-King, Sieberhagen, North: Paediatric nurse training activity in South Africa. A short report, forthcoming).

Original data collection was undertaken for Kenya, Malawi, Uganda and Zambia in the form of the questionnaire surveys and interviews described in Table 1. One respondent for each category was also included for South Africa to support consistency and corroboration of existing data. Data from all sources were then combined to provide a complete set of information for 2015, which was the only year for which a full set of data was available. The origins of all data are shown in the text and tables. In total, 15 individuals were invited to participate in the study. A summary of data sources is shown in Table 1.

**Table 1:** Overview of data sources and participation

	Kenya	Malawi	Uganda	South Africa	Zambia	Response
Respondent category (original data collection)						
Survey of children’s nursing leaders • Number of children’s nurses in practice • Nomenclature pertaining to this category of nurse • Number of training providers • Level at which qualifications awarded	•	•	•	•	•	5/5 (100%)
Survey of Heads of Schools of Nursing • Nature and duration of programmes offered • Minimum entry requirements • National and local policies or training plans • Number of graduates (2015)	•	•	n/a^a^	•	•	4/4 (100%)
Key informant interviews • Number of children’s nurses in practice nationally • Nomenclature pertaining to this category of nurse • Number of training providers	–	•	•	•	–	3/5 (60%)
Secondary sources						
Child Nurse Educators Forum records • Nature and duration of programmes offered • Number of graduates (2015)				•		7/7 (100%)
Chukwu 2017* • Nature and duration of programmes offered • Minimum entry requirements • National and local policies or training plans • Number of graduates (2012–2016)				•		5/7 (71%)
Total data sources						24

^a^Private correspondence indicates that this institution is not currently offering training relevant to the purposes of this study, with training having ceased around 2016–2017

*Source: [30] Key: • = Response invited and obtained. – = Response invited and not obtained

### Analysis and synthesis of data

Analysis of data was driven by the primary goal of this study, which was to identify as far as possible the extent of the children’s nursing workforce in five selected African countries. Quantitative data from questionnaire responses was collated and tabulated in the form of country-level findings. Data from interviews were transcribed by the researcher. An explicit transcription strategy and a consistent approach to coding content supported rigour during analysis [15]. Data derived from the processes described above were integrated or ‘mixed’ during analysis to generate as valid as possible a description of the capacity of the children’s nursing workforce in the five countries.

### Ethical approval

The study was approved by the Human Research Ethics Committee of the University of Cape Town (HREC REF: 411/2017).

## Results

Findings are presented in relation to reported children’s nursing workforce capacity, training activity and national training output. Analysis of qualitative data elicited two themes: the role of children’s nurses and their position within health systems, and the capacity of HRH information systems to accurately reflect the extent of the specialist children’s nursing workforce.

### Reported children’s nursing workforce capacity

The data collected suggest that the combined children’s nursing workforce reported for the five countries in this study totals 3728 nurses. South Africa accounts for around 8/10 of these nurses (3200, 84%). Table 2 presents reported children’s nursing workforce capacity. Data are presented alongside population estimates for babies and children 0–15 years to enable consideration of the numbers of children’s nurses relative to population.

**Table 2:** Reported children’s nursing workforce capacity (2015) relative to child population

	Kenya	Malawi	South Africa	Uganda	Zambia	Total
Children 0–15 years (% of total population)^a^	19.3 m (40.89%)	7.7 m (44.27%)	15.9 m (29.18%)	18.7 m (47.99%)	7.4 m (45.09%)	
Children’s nurses reported in practice^b^ (as % of total registered nurses)^c,d^	70 (0.09%)	92 (1.9%)	3115^ii^ (1.08%)	261 (1.58%)	105 (0.9%)	3728
Children’s nurses produced by training (2015)^b^	22	27	191^a^*^†^	0	32	272
Institutions training children’s nurses^b^	1	1	7*^†^	0	1	10
Programmes offered^b^	2	2	11*^†^	0	1	16

Sources:

^a^[31]

^b^Original data plus record of the Child Nursing Educator Forum Nov. 2017* and data from a postgraduate study (Chukwu, 2017)^†^

^c^[32]

^d^Annual circulars produced by the South African Nursing Council relating to Additional Qualifications on the Register between 2010 and 2016

Notes: ^i^It was subsequently confirmed that post-basic education leading to a registerable qualification in paediatric nursing is also offered by one further institution, although the relevant programmes were not offered during the period of data collection

^ii^South Africa was the only country for which information relating to the numbers of children’s nurses on the register was available through published information in the public domain

### Reported children’s nursing training activity

A total of 16 educational programmes leading to a qualification in paediatric nursing or child health nursing are offered by 10 institutions across the countries in this study, with Kenya, Malawi and Zambia having one institution each and South Africa hosting seven. Table 3 presents data relating to five countries where training is provided, or (in the case of Uganda) was provided until recently. One institution in Uganda was identified by a respondent as possibly offering relevant training, and attempts were made to contact this institution to participate in the survey of Heads of Schools of children’s nursing. No response was received. Private correspondence indicates that this institution is not currently offering training relevant to the purposes of this study, with training having ceased around 2016–2017.

**Table 3:** Reported children’s nursing training activity (Kenya, Malawi, Uganda, South Africa and Zambia)

Country	Institutions		Programmes				
	Count	Location	Title	Level^a^	Year est’d	Duration of study (years)	Graduates 2015
Kenya	1	Gertrude’s Children’s Hospital, Nairobi	Higher National Diploma in Pediatric Nursing	HND	2006	1	15
			Higher National Diploma in Pediatric Critical Care Nursing	HND	2013	1	7
Malawi	1	Kamuzu College of Nursing, University of Malawi	Bachelor of Science (Paediatric Nursing)	BSc	2010	3	6
			Master of Science in Child Health Nursing	MSc	2010	2	21
Uganda	0	Jinja School of Nursing and Midwifery (not currently being offered)	Diploma in Paediatric nursing	HND	–	–	–
South Africa	7	Ga-Rankuwa Nursing College, Pretoria, Gauteng	Post Basic Diploma in Child Nursing Science	6	–	1	22
		King Edward VII School of Nursing, Pietermaritzburg, KZN	Post Graduate Diploma Child Nursing Science	6	–	1	30
			PGDip Neonatal Nursing Science	6	–	1	30
		Lilitha Nursing College, East London, Eastern Cape	Post Basic Diploma in Child Nursing Science	6	–	1	14
		Rahima Moosa Nursing College, Johannesburg, Gauteng	Post Basic Diploma in Child Nursing Science	6	–	1	20
		University of Cape Town, Western Cape	Post Graduate Diploma in Child Nursing	8	2006	1	35
			Post Graduate Diploma in Child Critical Care Nursing	8	2009	1	16
			Masters of Nursing in Children’s Nursing	9	2015	2	–^b^
		Department of Nursing Science, University of Pretoria Gauteng	Bachelor of Nursing for Registered Nurses with Children’s Nursing Qualification	7	–	1	10
		University of the Free State School of Nursing, Bloemfontein, Free State	Advanced Diploma in Nursing (Child Psychiatric Nursing)	7	–	1	14
			Advanced Diploma in Nursing (Children’s Nursing)	7	–	1	
Zambia	1	Lusaka Schools of Nursing	Advanced Diploma in Pediatric Nursing	HND	2014	1	32
	10					Total	

^a^According to relevant national framework

^b^First graduates 2017

Notwithstanding the cessation of training in Uganda, the data suggest a steady growth in children’s nursing training activity since 2006 overall, measured by the number of programmes being offered. Prior to this, the data suggest that South Africa was the only country in southern Africa training children’s nurses. A total of eight new training programmes were reported to have been established between 2006 and 2016, in Kenya (in 2006 and 2013), Malawi (2010), Zambia (2014) and South Africa (2006, 2009 and 2015).

### National training output

Examination of the data in Table 4 suggests that a total of 81 children’s nurses were produced through training by Kenya, Malawi and Zambia in 2015 and 191 by South Africa for the same year. South Africa is the region’s largest children’s nursing training provider. Available data show that the number of children’s nurses reported trained at 5 out of 7 South African institutions between 2012 and 2016 equalled 60 (Chukwu, Shung-King, Sieberhagen, North: Paediatric nurse training activity in South Africa. A short report, forthcoming). Based on this, an estimated 179 children’s nurses are produced across South Africa’s seven training institutions on average per year.

**Table 4:** Estimation of in-country training output to 2015 (Kenya, Malawi and Zambia)

Country	Year training started	Number of years trainees produced (A)	Reported output 2015 (B)	Children’s nurse production to 2015 (A × B)
Kenya	2006	10	15	150
	2013	3	7	21
Malawi	2010	6	6	36
	2010	6	21	126
Zambia	2014	32	2	64
				Total produced by in-country training to 2015—397

Using the survey data obtained, an attempt was made to estimate training output to date for Kenya, Malawi and Zambia. As data were only available for a single year for these countries, the resulting estimations cannot be considered accurate, but are presented in Table 4 for illustrative purposes. Data suggest that by 2015 approximately 397 children’s nurses had been trained since programmes began in these three countries.

### Interviews

Interviewees confirmed the plausibility of the information obtained through questionnaires.

Two additional themes were identified.

#### The role of children’s nurses—perceptions of interviewees

Interviewees were asked to respond to the questions ‘How do children’s nurses contribute to children’s health in your country?’ and ‘Do children’s nurses carry out the same duties and tasks as other categories of nurses?’. They provided insightful descriptions of the role of children’s nurses and the way this category of nurse can contribute within lower resourced healthcare systems.

They contribute greatly in terms of how they are trained and the power to think and do the work they do. They are trained to identify conditions, make plans and commence the initial treatment before the doctor comes in. In Malawi, the ratio of doctors to patients is very low, and the patients are so many. Wherever they are working, these specialist nurses are empowered, through their specialist training…Because of that they are really able to take the lead in managing the children, depending on whichever areas they are in. In outpatients, they are able to triage children and say ‘this child requires quick attention’. They are able to facilitate the management. The delays associated with starting treatment are getting less now. (Nursing leader, Malawi)

The two [children’s] nurses at [name of hospital], they are very good. One is running the whole feeding clinic. (Nursing leader, Uganda)

Specialist children’s nurses should mostly be working at the level of district hospitals. Then you need sub-specialists at regional and tertiary level – the neonatal nurses, and critical care [children’s] nurses. And the role of children’s nurses [at district hospital level] is to be the institutional memory. They are the hearts and brains of a service. (Child health leader, South Africa)

#### Capacity of information systems—perceptions of interviewees

Interviewees identified the growth of specialisation as having implications for information systems: ‘The growth of specialists is a new demand on the register. In Malawi now we have those doing nursing education, reproductive health, midwifery, community... It’s a different dynamic and we need to respond accordingly, as a country and as a registering board. The recording is done according to qualifications. So if a nurse has a masters in paediatrics, they are recorded as having such. [The register] records the qualification held, but it does not separately record the category of specialists. We are still yet to put up a category of all of the specialists.’ (Nursing leader, Malawi)

The information provided by interviewees concurred with the finding that only South Africa routinely monitors and reports on the size of the registered children’s nursing workforce currently. Even in this case, one interviewee expressed the view that this information is not being used to inform planning: ‘The main focus … is on norms and numbers [of all categories of nurses], it’s not about specialisation.’ (Interview participant, South Africa)

One interviewee suggested that the benefits of information specific to the children’s workforce may not be understood by decision makers due to a lack of understanding of the role: ‘The contribution of the specialist children’s nurses that do exist isn’t always made the most of in the services, they [the services] don’t have that understanding.’ (Interview participant, Uganda)

### Existence of polices guiding training

Heads of Schools of Nursing responding to the questionnaire survey and key informants participating in telephone interviews were asked about the existence of national and local policies or training plans determining the provision of children’s nursing training. Very minimal information was elicited. Participants were asked ‘Are there national and local policies or training plans which guide the provision of children’s nursing training in your institution/country?’ Respondents mentioned a total of 11 sources of guidance relevant to the provision of children’s nursing training relating to Malawi, South Africa and Zambia. Of these, four were not possible to identify for review (e.g. ‘the child health policy’). Seven were assessed as not relevant for review (e.g. the document took the form of a specific clinical practice guideline, e.g. Helping Babies Breathe). One item was identified as relevant and was reviewed. This was the South African Nursing Council’s specification of competencies for the category of specialist children’s nurse (South African Nursing Council, 2012). The existence and nature of national policies and strategies guiding the development of the children’s nursing workforce requires further investigation.

### Limitations

The lack of response from national nursing councils meant that an important data source intended to enable triangulation was omitted. The information could not be identified through Internet searching of published records of the remaining national nursing councils, either because registers are not available for inspection or because children’s nursing qualifications could not be extracted from published totals. South Africa was the only country for which information relating to the numbers of children’s nurses on the register was available through published information in the public domain.

The decision to collect data relating to training outputs for a single year (2015) was made to reduce the burden on respondents. Confining data to a single year limited the ability to calculate an accurate total for training output, as reported 2015 output may not be typical.

### Reliability and validity

No substantial inter-respondent disparities were apparent in respect of the data for any of the five countries. Triangulation through telephone interviews corroborated survey data.

Data were further assessed for internal consistency as follows for each country: (a) estimation of annual output through training based on reports for 2015, (b) multiplication of that figure by the number of years the course(s) have been in operation, and (c) the resulting number should be equal to or less than the number of children’s nurses on the register or reported to be in practice. It is recognised that student numbers may vary year to year in reality.

In the absence of participation by the South African Nursing Council, the reliability of the figure reported for South Africa (3200) was assessed through analysis of the annual reports produced by the South African Nursing Council relating to Additional Qualifications on the Register listed between 2010 and 2016 (3115) (South African Nursing Council, 2010–2016). It was not possible to perform this check for the other countries, either because registers were not available for inspection or because children’s nursing qualifications could not be disaggregated from published totals.

## Discussion

The introduction of specialist children’s nurses into these health systems is still at an early stage, and training activity is on a small scale with only South Africa having more than one training centre. Three of the five countries began training children’s nurses less than 5 years ago. It is to be expected therefore that at this stage, the numbers of children’s nurses will be extremely small and inadequate to meet population needs. These limited data, obtained in the absence of reliable official information, identify for the first time a small but growing cohort of specialist children’s nurses within the five countries, supported by relatively recently established training activity in four of the five countries. South Africa has the largest training output, producing 179 children’s nurses per year on average. Average annual training output for the other four countries combined is 81 children’s nurses, meaning that total output across the five countries adds 260 children’s nurses to the workforce on average per year. Given the investment in health workforce development this represents, there is a need to consider how HRH information systems can best support informed decision making.

### Investing in developing the children’s nursing workforce

The actions being taken to develop children’s nursing capacity identified through this study suggest that health service decision-makers in these countries believe that, in the context of a widening gap between the growing needs of an expanding child population and the ability of health systems to meet these needs, one justifiable response is to increase the capacity of the children’s nursing workforce. The impact on the wage bill of employing different groups of worker varies considerably. As Scheffler highlights, physicians are expensive, nurses somewhat less so, and lay health workers cheaper still [16]. At the same time, pre-service (basic) training, and by extension post-basic training, has to be seen as a long-term solution as it takes time for specialist nurses to reach their full capacity within the system [17]. In the context of the lower-resourced, demand-stretched healthcare systems considered by this study, the training activity documented represents a strategically significant investment in workforce development.

The composition as well as the size of the health workforce is an important consideration when seeking to manage health workforce shortages, requiring a focus on more than simply ‘strength in numbers’ [16, 18]. Specialist nurses may offer a cost-effective way to maximise the effectiveness of the health workforce in Africa, but intelligent development and deployment of this group of staff requires accurate workforce data to be available. It also requires decision makers to possess a clear strategic vision regarding the contribution of these nurses within a nation’s health system.

It is evident that the number of children’s nurses is extremely small in relation to the extensive child population. Taking Malawi as an example, the ratio of children’s nurses to children aged 0–15 is approximately one nurse per 83 696. Children’s nurses make up 1.9% of nurses on the register in Malawi, compared with 7% of nurses on the register in England [19]. It would be preferable to consider the data in relation to staffing levels and patterns of demand for care in paediatric wards and health facilities, but the ability to estimate workforce needs accurately for specialist nursing cadres in LMICs requires further development.

### The envisaged role of children’s nurses in African health systems

Respondents in this small sample were able to articulate very clearly the type of contribution that children’s nurses make within their health systems. There is a need to better understand the rationale and the motivations behind the growth of training activity identified through this study. However, respondents’ comments suggest that children’s nurses in Africa’s health systems perform a wide variety of clinical, professional and organisational leadership roles, some of which have potentially far-reaching impacts on children’s health services and systems. The United Nations high-level commission on health employment and economic growth, with input from the International Council of Nurses, has articulated the need for a comprehensive approach to nursing workforce development, concluding that capacity building efforts must address both quantity and quality by developing nurses’ capacity to practise high-quality care; educate and train other health workers; lead effective teams, organisations and systems; and advocate for health in partnership with others [18].

The roles and contributions described by interviewees align with calls for investment in personal resilience and leadership capacity [20], quality of care and the capacity of clinical leaders to teach, support and manage [21, 22], at the same as increasing staff numbers. This is consistent with the global vision for nursing and midwifery development articulated by the World Health Organization, which sees an appropriately skilled nursing workforce as providing a cost-effective and efficient intersection for inter- and intra-disciplinary health actions, with the nursing workforce amplifying efforts to strengthen healthcare systems [2].

While the WHO’s global strategy points to a need for the nursing workforce to possess the skills and competencies required to serve specific population needs, there remains a need to better define what these skills and competencies are at a regional and country level across the countries in this study.

### Monitoring the children’s nursing workforce

Specialist nurses may offer a cost-effective way to maximise the effectiveness of the health workforce in Africa, but intelligent development and deployment of this group of staff is only possible if accurate workforce data is available. Many policies dealing with the nursing workforce tend to assume that all nurses have similar qualifications and that it is efficient to deploy them interchangeably [23].

However, the information generated through this study regarding the extent of the registered children’s nursing workforce suggests that country-level HRH information systems continue to consider the nursing workforce as comprising generalists, with little apparent consideration of specialisation. Recording of the nursing workforce cannot sensibly be approached as though all nurses are generalists.

### Limitations

None of the national nursing registries invited to participate in the study provided the information requested about the composition of the registered children’s nursing workforce and the approach to recording. Published information relating to the composition of the registered children’s nursing workforce was available for only one country (South Africa), based on statutory records acknowledged by the national Department of Health as ‘not a true reflection of the numbers of health professionals available for the workforce’ [14]. No attempt was made to establish, in relation to the numbers of children’s nurses reported, how many were active in the workforce and how many of those were working in paediatrics or child health. These questions will form the basis of further research.

### The capacity of HRH information systems to monitor the children’s nursing workforce

There are widely acknowledged concerns regarding the capacity of nursing regulatory bodies to monitor and report on the nursing workforce in the countries concerned [6, 24, 25], and the literature suggests that nursing regulatory bodies in Kenya [26] and Uganda [27–29] appear to have participated in targeted capacity building interventions focusing on HRH information systems.

The Capacity Project provides an account of the experience of the individuals involved in developing a new HRH information system at Uganda’s national nursing registry, in a vividly descriptive report titled ‘I can now speak boldly’ [28]. The report speaks to feelings of deep responsibility on the part of regulators, a sense of guilt at the inadequacy of systems to respond to the need for information, as well as a wish to improve the situation. When effective systems were successfully implemented, registry staff reported satisfaction and professional pride.

While the reasons for non-participation by nursing regulatory bodies in this study are not known, it is possible that situations similar to the one described above may have influenced the decision of officials not to respond. This suggests that appropriately contextualised collaborative interventions to improve capacity to monitor the specialist registered children’s nursing workforce at country levels could be welcomed.

## Conclusions

The efforts directed towards establishing a specialist children’s nursing workforce reported in this study point towards a need for HRH information systems to change in order to provide a sound basis for decisions about workforce composition, workforce development and training plans. The findings of this initial attempt to provide an estimation of the extent of the children’s nursing workforce in five selected African countries suggest that further action is needed to achieve a more finely grained understanding of the state of the specialist children’s nursing workforce, so that this growing population of children’s nurses can be developed and utilised to best effect within Africa’s health systems.
